# Adhesion-driven vesicle translocation through membrane-covered pores

**DOI:** 10.1016/j.bpj.2025.01.012

**Published:** 2025-01-24

**Authors:** Nishant Baruah, Jiarul Midya, Gerhard Gompper, Anil Kumar Dasanna, Thorsten Auth

**Affiliations:** 1 Theoretical Physics of Living Matter, Institute of Biological Information Processing and Institute for Advanced Simulation, Forschungszentrum Jülich, Jülich, Germany; 2 School of Basic Sciences, Indian Institute of Technology, Bhubaneswar, India; 3 Department of Theoretical Physics and Center for Biophysics, Saarland University, Saarbrücken, Germany; 4 INM–Leibniz Institute for New Materials, Saarbrücken, Germany; 5 Department of Physical Sciences, Indian Institute of Science Education and Research Mohali, Knowledge City, Manauli, India

## Abstract

Translocation across barriers and through constrictions is a mechanism that is often used in vivo for transporting material between compartments. A specific example is apicomplexan parasites invading host cells through the tight junction that acts as a pore, and a similar barrier crossing is involved in drug delivery using lipid vesicles penetrating intact skin. Here, we use triangulated membranes and energy minimization to study the translocation of vesicles through pores with fixed radii. The vesicles bind to a lipid bilayer spanning the pore, the adhesion-energy gain drives the translocation, and the vesicle deformation induces an energy barrier. In addition, the deformation-energy cost for deforming the pore-spanning membrane hinders the translocation. Increasing the bending rigidity of the pore-spanning membrane and decreasing the pore size both increase the barrier height and shift the maximum to smaller fractions of translocated vesicle membrane. We compare the translocation of initially spherical vesicles with fixed membrane area and freely adjustable volume to that of initially prolate vesicles with fixed membrane area and volume. In the latter case, translocation can be entirely suppressed. Our predictions may help rationalize the invasion of apicomplexan parasites into host cells and design measures to combat the diseases they transmit.

## Significance

Lipid-bilayer membranes compartmentalize biological systems. Similar to translocation through pores, membrane budding controls the exchange of material and information between the compartments. In previous work, an osmotic-pressure difference has been used to drive vesicle-pore translocation; we study translocation driven by the vesicle adhering to a host membrane. Using computer simulations, we predict free, partial-translocated, and complete-translocated states. Our work may help to understand the wrapping of vesicles at plasma membranes supported by a cortical cytoskeleton and the invasion of apicomplexan parasites into their host cells.

## Introduction

In vivo, vesicles are abundant carriers for transporting material within and between cells. Examples are synaptic vesicles in neurotransmission (1), extracellular vesicles that are involved in physiological processes and proposed as drug-delivery vehicles (2), synthetic liposomes for drug delivery (3), and enveloped viruses, such as severe acute respiratory syndrome coronavirus-2 (SARS-CoV-2) (4,5). Vesicles that deliver their content to host cells often fuse with the host plasma membranes and thereby directly deliver their material to the membrane and the cytosol (6,7). However, endocytosis of entire vesicles at the plasma membrane is also an important uptake mechanism for extracellular vesicles (8) and enveloped viruses (9). Because mammalian cells usually feature a cortical cytoskeleton below their plasma membrane, vesicles that are being endocytosed may need to “squeeze” through the cytoskeletal network; for example, the typical mesh size of the spectrin cytoskeleton of human erythrocytes is 60−100nm (10).

Adhesion, wrapping, and squeezing have been hypothesized to be relevant for the entry of apicomplexan parasites into their parasitophorous vacuoles within the host cells (11,12,13): both *Plasmodium* and *Toxoplasma* deform upon invading host cells and squeeze through a “tight junction” (14,15), which appears as an electron-dense zone in microscopy but whose architecture is not yet entirely understood. In *Toxoplasma*, an actin ring within the parasite is found at the constriction (14). For mammalian host cells, the deformation energy of the cortical cytoskeleton, a polymerized membrane with fixed connectivity of the polymers, additionally suppresses complete engulfment (16,17). Therefore, for successful invasion of *Plasmodium* into human erythrocytes, a local disassembly of the cortical spectrin cytoskeleton has been hypothesized (18); the surrounding intact cytoskeleton might constrict the parasite during invasion.

The translocation of vesicles through pores has been studied using theory and computer simulations for various systems that differ mainly by the driving force for translocation and the membrane’s elastic properties. Many studies have been motivated by drug delivery in the skin where an osmotic-pressure difference drives the translocation (19). For partial-translocated initially spherical vesicles, the vesicle membrane deformation energy increases compared to free vesicles, and the vesicle volume decreases (20). For identical pore radii, a pore with a finite length increases the translocation-energy barrier compared to a pore with a vanishing length because of the increased compression of the vesicle (21). An exponential decay of the translocation time with increasing driving force has been predicted for fluid vesicles, whereas a power-law dependence is expected for polymerized vesicles (21,22). A power-law dependence has also been reported for the critical strength of a homogeneous field driving pore translocation of fluid vesicles (23). Although all vesicle studies discussed above assume a fixed membrane area and a variable vesicle volume, vesicles with fixed volume and variable area—where the membrane stretching energy dominates—–have also been studied (24).

**Figure 1:**
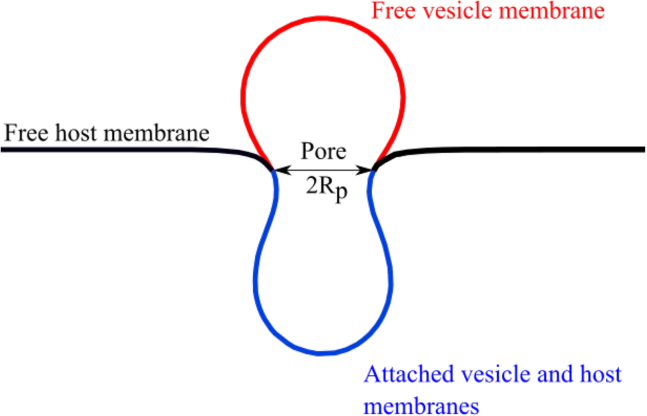
Initially spherical vesicle of radius $R_{v}$ translocating through a circular pore of radius $R_{p}=R_{v}/2$ spanned by a host membrane with bending rigidity $\kappa_{h}$ and tension $\gamma R_{v}^{2}/\kappa_{h}=50$. The snapshot corresponds to the vesicle-membrane translocation fraction ρ=0.5.

Here, we study the translocation of vesicles through circular, membrane-covered pores (see Fig. 1). We compare our predictions for initially spherical vesicles with fixed membrane areas and variable volumes with those for prolate vesicles with fixed membrane areas and fixed volumes. Note that in a physiological environment, the osmotic concentrations are high; therefore, free vesicles can have a fixed reduced volume v<1 and a nonspherical shape (25). Volumes and membrane areas are chosen to correspond to those of *Plasmodium* and *Toxoplasma*. Within the pore, the vesicles adhere to the membrane covering the pore, providing an adhesion-energy gain that drives translocation. Using a continuum-membrane model, we calculate energy landscapes for vesicle translocation and determine translocation states and times. The deformation-energy costs for the pore-spanning membrane hinder translocation, leading to an energy-barrier maximum where less than half of the vesicle area has translocated. For large pores, the energy barrier can be considerably lower for prolate vesicles than for initially spherical vesicles without a target volume, resulting in translocation times that are orders of magnitude shorter. For small pores, prolate vesicles with fixed areas and volumes may not translocate through the pore at all.

First, we introduce the model and compare translocation-energy landscapes obtained using a spherical-cap geometry and triangulated membranes. In the results sections, we calculate stable translocation states, energy barriers, and translocation times for initially spherical and prolate vesicles. We characterize the dependence of the translocation transitions on membrane curvature-elastic parameters, pore and vesicle sizes, and vesicle adhesion strengths. Finally, we summarize our results and discuss their relevance for the invasion of deformable apicomplexan parasites into their host cells.

## methods

We study the translocation of a vesicle through a pore modeled by a circular ring of fixed radius embedded in a fluid membrane (see Fig. 1). In our calculations, the contact line between the vesicle and the pore-spanning membrane can have a wider radius than the pore; however, it coincides with the pore for all cases we have studied; see supporting material. The deformation-energy costs of the vesicle and the pore-spanning membrane hinder translocation, whereas the adhesion-energy gain for the contact between the vesicle and the pore-spanning membrane drives translocation. A comparison of the adhesion-driven translocation with osmotic-pressure driven translocation, as assumed, e.g., in Refs. (20,21), is provided in the supporting material.

**Figure 2:**
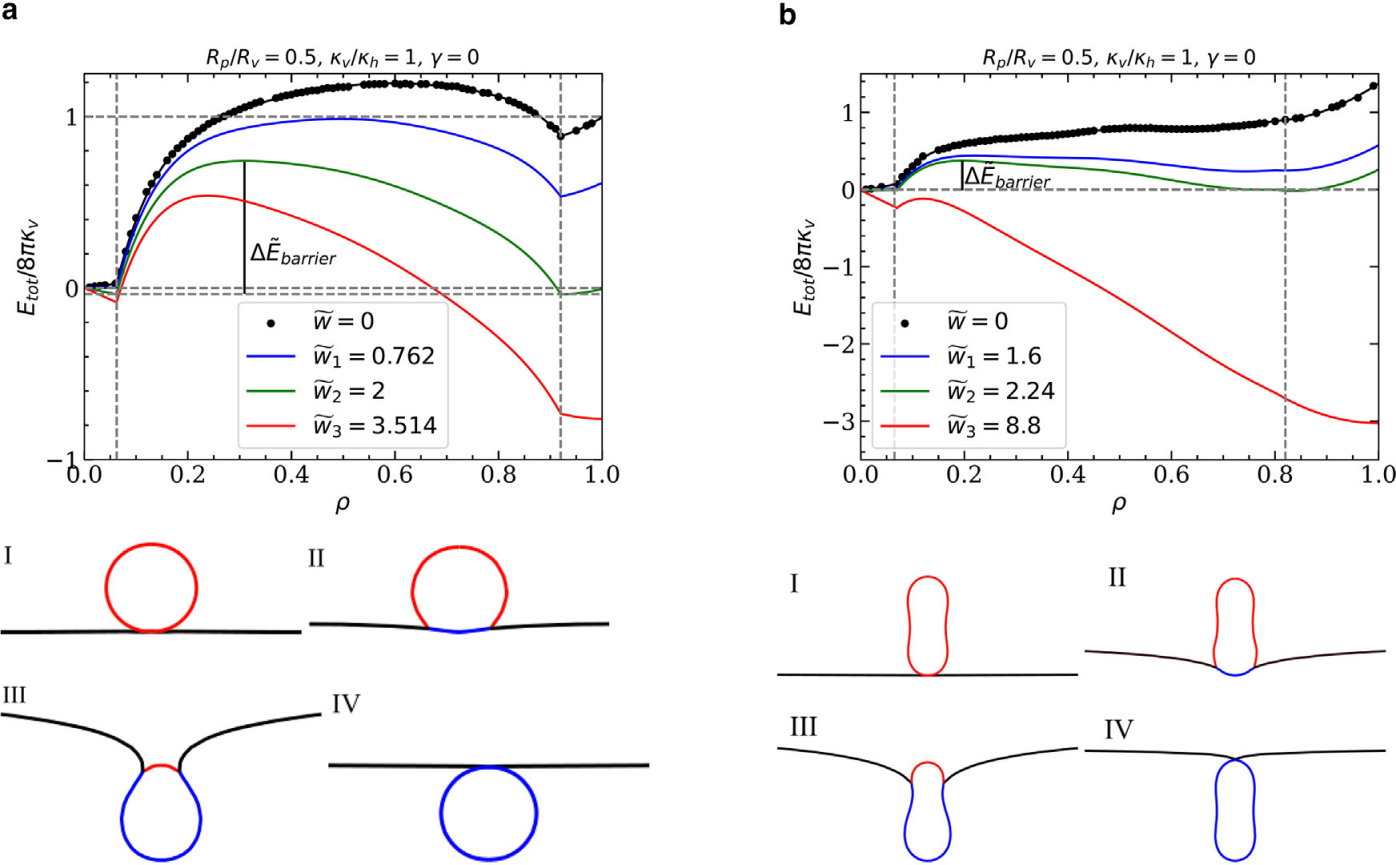
Energies and simulation snapshots for a vesicle-pore system with $R_{p}/R_{v}=0.5$ and $\kappa_{v}/\kappa_{h}=1$ as a function of the fraction ρ of translocated vesicle membrane area for (*a*) an initially spherical vesicle, and (*b*) a prolate vesicle with reduced volume v=0.8. Black points indicate the deformation energies calculated using triangulated membranes, black lines piecewise fits of the data. Vertical dashed lines indicate the range of translocation fractions where the vesicle touches the rim of the pore. The snapshots show free-vesicle (I), weak- (II), and deep- (III), and complete-translocated states (IV). The reduced adhesion strengths are ${\tilde{w}}_{1}$ for the transition I → II, ${\tilde{w}}_{2}$ for the transition II → III, and ${\tilde{w}}_{3}$ for the transition III → IV, with $\tilde{w}=wR_{v}^{2}/\kappa_{v}$. The reduced energy barrier for the pore-passage transition II → III is $\Delta {\tilde{E}}_{\text{barrier}}=E_{\text{barrier}}/\left(8\pi \kappa_{v}\right)$.

We calculate the total deformation energy and the translocation-energy contributions using a continuum-membrane model,

(1) $$\begin{array}{ccc} E & = & 2\kappa_{v}\int_{S_{v}}dS\;{\left(H-c_{0}\right)}^{2}+\int_{S_{h}}dS\;\left(\gamma +2\kappa_{h}H^{2}\right)\\ & & -w\int_{S_{\text{ad}}}dS+\gamma_{v}S_{v}+p_{v}V_{v}\text{,} \end{array}$$

see Fig. 2. Here, $H=\left(c_{1}+c_{2}\right)/2$ is the mean curvature, and $c_{1}$ and $c_{2}$ are the principal curvatures at each point of the membrane. The integrals are calculated over the entire membrane areas $S_{v}$ of the vesicle and $S_{h}$ of the host; $S_{\text{ad}}$ indicates the area of the double-bilayer of the bound vesicle and host membranes. The membrane’s curvature-elastic properties are characterized by the bending rigidities $\kappa_{v}$ of the vesicle and $\kappa_{h}$ of the host; in addition, the vesicle membrane may be subject to a spontaneous curvature $c_{0}$ and the host membrane to a tension γ. The vesicle-host contact interaction, which is applied within the pore only, is characterized by the adhesion strength w.

If applicable, the Lagrange multipliers $\gamma_{v}$ and $p_{v}$ fix the vesicle’s membrane area $S_{v}$ and volume $V_{v}$, respectively. We study 1) initially spherical vesicles with fixed membrane area, $\gamma_{v}\neq 0$, and freely adjustable volume, $p_{v}=0$; and 2) initially prolate vesicles with both a fixed area and volume, $\gamma_{v}\neq 0$ and $p_{v}\neq 0$. The volume of the initially spherical vesicles decreases during translocation compared with the volume in the free state (see supporting material). The shapes of the initially prolate vesicles are characterized by the reduced vesicle volume

(2) $$v=6\sqrt{\pi }V_{v}/S_{v}^{3/2}\text{.}$$

The tension and pressure to achieve the target values of membrane area and vesicle volume depend on the translocation fraction *ρ* (see supporting material).

The basis for all predictions of our model is the calculation of the total energy of the system for various fractions $\rho =S_{\text{ad}}/\left(S_{\text{free}}+S_{\text{ad}}\right)$ of the vesicle’s membrane area having translocated through the pore (see Figs. 1 and 2). We first calculate the deformation energies only (w=0). For simplicity, we assume $c_{0}=0$, although a finite spontaneous curvature affects particle wrapping by fluid membranes (26,27) and may be induced in vivo, for example, by membrane-bound proteins (28,29). For initially spherical vesicles, the deformation-energy landscape can be estimated using mostly analytical calculations based on a spherical-cap model (see Appendix A and supporting material). More accurate energies are obtained using triangulated membranes (30,31) and energy minimization to predict equilibrium shapes and energies; triangulated-membrane calculations are required for initially prolate vesicles. For triangulation and energy minimization, we employ the freely available program “Surface Evolver” (32); details of the minimization algorithm are provided in Appendix B.

In the following, we characterize the vesicle size using the radius $R_{v}$ of a sphere with the same surface area as the vesicle and the size of the circular pore using its radius $R_{p}$.

### Characterizing translocation-state transitions

The analysis of total energies for various adhesion strengths is performed analogously to the analysis for wrapping particles at membranes (33,34). Assuming a contact interaction between vesicles and host membranes, the adhesion-energy gain is proportional to the adhered membrane area and, thus, to the translocated fraction of the vesicle membrane; it is added to the deformation energy costs. With the help of determining minima, maxima, and vanishing slopes in the deformation-energy landscape, transitions between stable translocation states, energy barriers, and translocation times are predicted. The triangulated-membrane data for the deformation energy are fit using a piecewise function f(ρ) that consists of fourth-order polynomials; deformation energies obtained using the spherical-cap model are analyzed directly.

In general, we find the existence of four stable states in which the system can reside: in state I, the vesicle body has not contacted the host membrane; in state II, the vesicle is attached to the membrane, but most of its membrane area has not translocated through the pore; in state III, the majority of the vesicle membrane area has translocated, but the vesicle is not yet entirely enveloped by the host membrane; in state IV, the vesicle has translocated completely and is completely enveloped by the host membrane. We refer to the transitions I → II, II → III, and III → IV as binding, pore-passage, and envelopment transitions, respectively. The translocation-energy landscapes for initially spherical and initially prolate vesicles differ significantly (see Fig. 2).

Our study combines pore translocation with adhesion and wrapping. For the binding transition, the vesicle does not yet directly interact with the pore. The adhesion strengths for the binding transition agree with those for vesicle wrapping without the presence of a pore (35,36), and the deformation energies calculated using triangulated membranes and the spherical-cap model are very similar. Similarly, the vesicle does not directly interact with the pore near complete translocation (wrapping), and the deformation energies are the same as for a wrapping-only system (35).

The shapes of vesicles that are constricted by the pore are strongly deformed compared with their free-vesicle shapes. The deformation energies increase steeply with increasing translocation fraction and experience a maximum (see Fig. 2), rendering partial-wrapped states unstable in a wide range of translocation (wrapping) fractions that are stable in a wrapping-only system (35,36). The translocation-energy barrier for the pore-passage transition is characterized by the difference between the maximum and the minima at adhesion strengths w for that the minima before and after pore-passage have equal energies (see Figs. 4 and 7), as well as the translocation fractions for the maximum and the minima (see supporting material). For the same ratio of pore and vesicle radii, we find much lower energy barriers for the “thinner” initially prolate vesicles than for initially spherical vesicles (see supporting material). In both cases, the energy-barrier maximum is found for less than half translocation. The energy landscape shows a kink as a clear signature of detaching from the pore in the case of initially spherical vesicles; this feature is missing for prolate vesicles.

## Translocation of initially spherical vesicles

**Figure 3:**
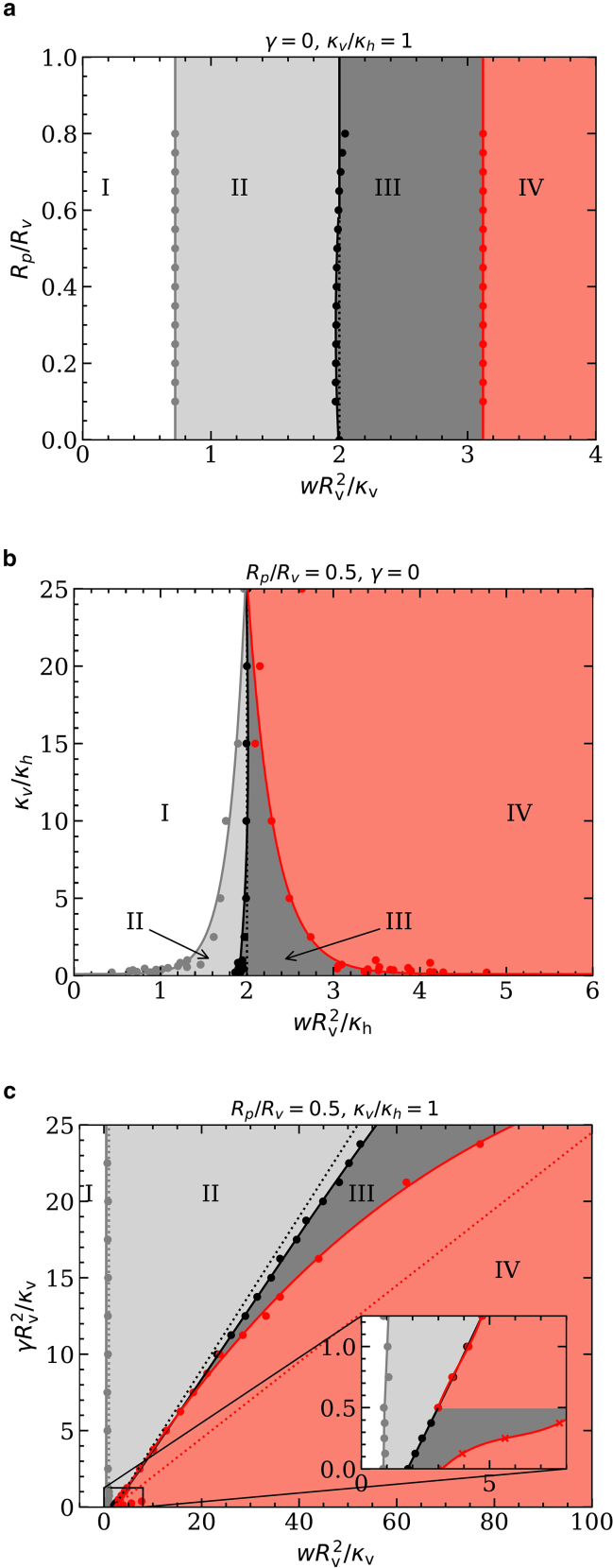
Translocation-state diagrams for initially spherical vesicles for (*a*) fixed $\kappa_{v}/\kappa_{h}=1$, various $R_{p}/R_{v}$ and adhesion strengths $wR_{v}^{2}/\kappa_{v}$; (*b*) fixed $R_{p}/R_{v}$, various $\kappa_{v}/\kappa_{h}$ and adhesion strengths $wR_{v}^{2}/\kappa_{h}$; and (*c*) fixed $R_{p}/R_{v}$, $\kappa_{v}/\kappa_{h}=1$, various membrane tensions $\gamma R_{v}^{2}/\kappa_{v}$ and adhesion strengths $wR_{v}^{2}/\kappa_{v}$: free-vesicle (I), weak- (II), deep- (III), and complete-translocated (IV) state. Spherical-cap model, dotted lines; triangulated membranes, points and solid lines.

Initially spherical vesicles can squeeze through arbitrarily small circular pores. However, unless the pore size is similar to the vesicle size, a typical energy barrier from a weak- to a deep-translocated state has a height of the order of $8\pi \left(\kappa_{v}+\kappa_{h}\right)$ (see Fig. 2 *a*). Fig. 3 shows translocation-state diagrams indicating the stable states for various pore-to-vesicle size ratios, vesicle-to-host-membrane bending-rigidity ratios, and host-membrane tensions. Most state boundaries predicted using triangulated membranes agree well with those obtained using the spherical-cap model—except for the envelopment transition at finite host-membrane tension.

### Stable translocation states

At small adhesion strengths, the vesicles do not adhere to the host membranes. Assuming that they bind first in the centers of the pores, the binding transition is continuous and occurs at adhesion strengths known from vesicle-wrapping calculations (36). For hard spherical particles at tensionless membranes, the deformation energies increase linearly with increasing wrapping fraction because of their homogeneous surface curvature (33,34,37). Vesicles flatten where they bind to the host membrane, which decreases the total system energy for shallow-wrapped states. Therefore, decreasing $\kappa_{v}/\kappa_{h}$ facilitates vesicle binding and initial wrapping (36,38) (see Fig. 3 *b*). The adhesion strength for the binding transition is independent of the pore-to-vesicle size ratio $R_{p}/R_{v}$ and the host-membrane tension γ (see Fig. 3 *a* and *c*).

While squeezing through the pore, the vesicle deformation because of the constriction induces an energy barrier. Therefore, pore-passage transitions are always discontinuous and between a weak- and a deep-translocated state. In the limit of vanishing pore size, the transition occurs directly between the free and the complete-translocated states, for γ=0 at $wR_{v}^{2}/\kappa_{h}=2$. The adhesion strength for the pore-passage transition depends only weakly on $R_{p}/R_{v}$ and $\kappa_{v}/\kappa_{h}$ (Fig. 3 *a* and *b*). Interestingly, we find a direct transition between the free and the complete-translocated state for high bending-rigidity ratios $\kappa_{v}/\kappa_{h}$ (see Fig. 3 *b*). However, a significant amount of host-membrane area is required for vesicle wrapping. Therefore, the host-membrane deformation-energy difference between deep- and weak-translocated states and, thus, also the adhesion strength for the pore-passage transition both strongly increase with increasing host-membrane tension (see Fig. 3 *c*).

The vesicle-envelopment transition completes the pore translocation process. Because vesicle deformation stabilizes partial-translocated states, decreasing $\kappa_{v}/\kappa_{h}$ impedes complete translocation; the envelopment transition shifts to higher adhesion strengths yet remaining finite for $\kappa_{v}/\kappa_{h}\to 0$ (see Fig. 3 *b*). For vanishing host-membrane tension γ=0, the envelopment transition is continuous, and the vesicle has already detached from the rim of the pore. Therefore, the adhesion strength for the transition is independent of the pore-to-vesicle size ratio $R_{p}/R_{v}$.

With increasing host-membrane tension γ, the envelopment transition shifts to significantly higher adhesion strengths (see Fig. 3 *c*). Although this is qualitatively also predicted by the spherical-cap model, the latter significantly overestimates the value of the adhesion strength. The triangulated-membrane data show a very complex translocation-state behavior for finite tensions near complete translocation. A continuous envelopment transition from a deep- to the complete-translocated state is found below a threshold tension $\gamma R_{v}^{2}/\kappa_{v}\approx 0.5$. Increasing the tension further, we first observe a direct and discontinuous transition between the shallow-translocated and the complete-translocated state, with the deep-translocated state being metastable. For $\gamma R_{v}^{2}/\kappa_{v}≳10$, the deep-translocated state is again stable, but the envelopment transition is discontinuous. Although, at small tensions, the stable deep-wrapped state is egg shaped, it assumes a pronounced pear shape in the stable deep-wrapped state at high tensions.

### Energy barriers

**Figure 4:**
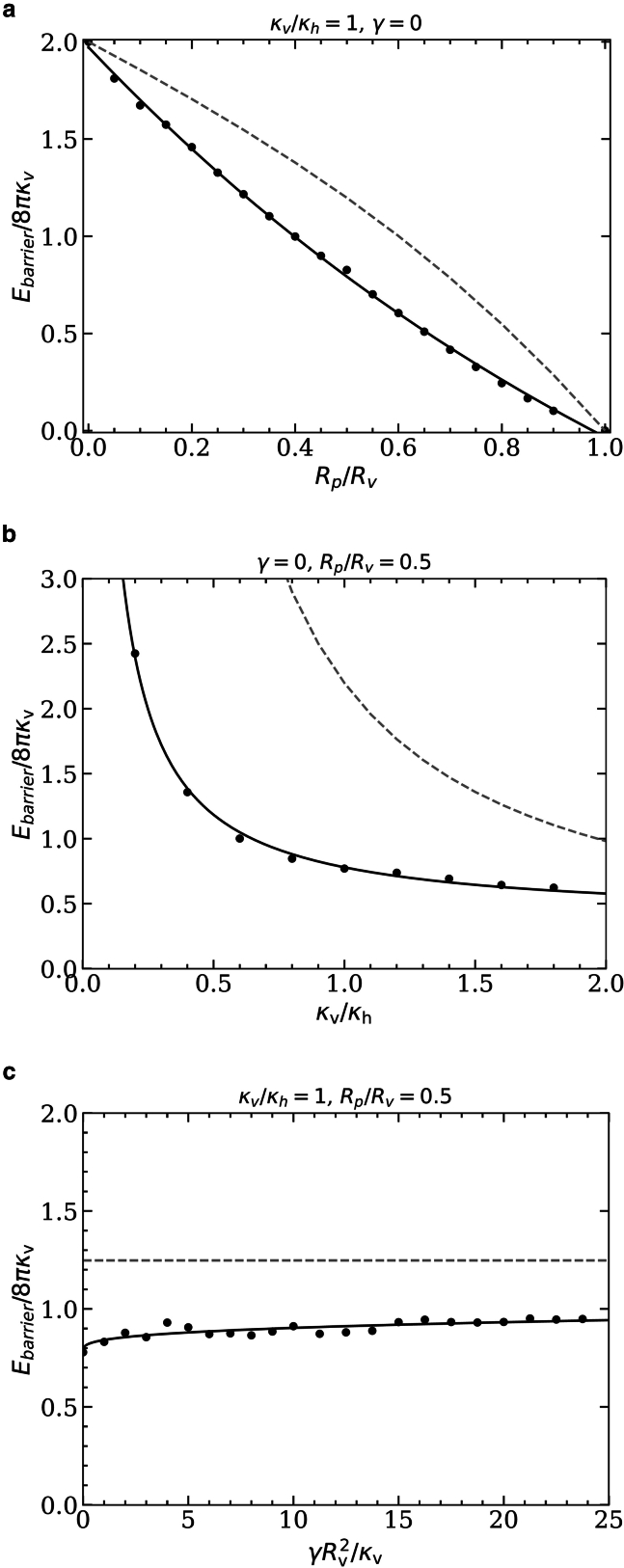
Energy barriers for the pore-passage transition of initially spherical vesicles and (*a*) $\kappa_{v}/\kappa_{h}=1$, γ=0, and various vesicle-pore size ratios $R_{p}/R_{v}$; (*b*) γ=0, $R_{p}/R_{v}=0.5$, and various vesicle-host bending-rigidity ratios $\kappa_{v}/\kappa_{h}$; and (*c*) $\kappa_{v}/\kappa_{h}=1$, $R_{p}/R_{v}=0.5$, and various host-membrane tensions γ. Data are shown for the spherical-cap model (*dashed lines*) and triangulated membranes (*points*); the fit functions for the triangulated-membrane data are provided in the SI.

Fig. 4*a*–*c* show the energy barriers for the pore-passage transitions for various pore-to-vesicle size ratios, vesicle-to-host-membrane bending-rigidity ratios, and host-membrane tensions, respectively, compared with Fig. 2. In all cases, the energy barriers obtained using the triangulated-membrane model are lower than those obtained using the spherical-cap model. This is expected because the significantly higher number of degrees of freedom for the triangulated-membrane model compared with the spherical-cap model yields energetically more favorable vesicle shapes for partial-translocated states.

With increasing pore-to-vesicle size ratio $R_{p}/R_{v}$, the energy barrier decreases because the vesicle needs to deform less to squeeze through the pore (see Fig. 4 *a*). The energy barrier vanishes for $R_{p}/R_{v}=1$, where the vesicle can translocate (almost) without being deformed by the pore. The energy barrier is maximal for vanishing pore size, where the bending energy increases by $8\pi \left(\kappa_{v}+\kappa_{h}\right)$ for an infinitesimally small translocation fraction. For small values of $\kappa_{v}/\kappa_{h}$, the deformation of the host membrane contributes significantly to the energy barrier, whereas for high values of $\kappa_{v}/\kappa_{h}$ the barrier is dominated by the bending rigidity $\kappa_{v}$ of the vesicle membrane, using both the spherical-cap and the triangulated-membrane model (see Fig. 4 *b*). In the latter regime, the energy barrier is proportional to the bending rigidity $\kappa_{v}$ of the vesicle. With decreasing vesicle bending rigidity, the energy barrier will eventually depend on the host-membrane bending rigidity $\kappa_{h}$ only.

Interestingly, the energy barrier increases only weakly with increasing host-membrane tension $\gamma R_{v}^{2}/\kappa_{v}$ for the triangulated-membrane model and is independent of the tension for the spherical-cap model (see Fig. 4 *c*).

### Translocation times

We calculate translocation times using the Fokker-Planck equation for the “diffusion” of vesicles across the energy barrier at finite vesicle-host adhesion strengths w. For a Brownian particle subject to thermal motion, the translocation time is estimated based on the probability distributions for finding the particle at specific locations in the energy landscape (21),

(3) $$\tau =\frac{1}{k_{0}}\int_{0}^{1}d\rho_{1}\int_{0}^{\rho_{1}}d\rho_{2}\;\exp\left[\frac{E\left(\rho_{1}\right)-E\left(\rho_{2}\right)}{k_{B}T}\right]\text{.}$$

**Figure 5:**
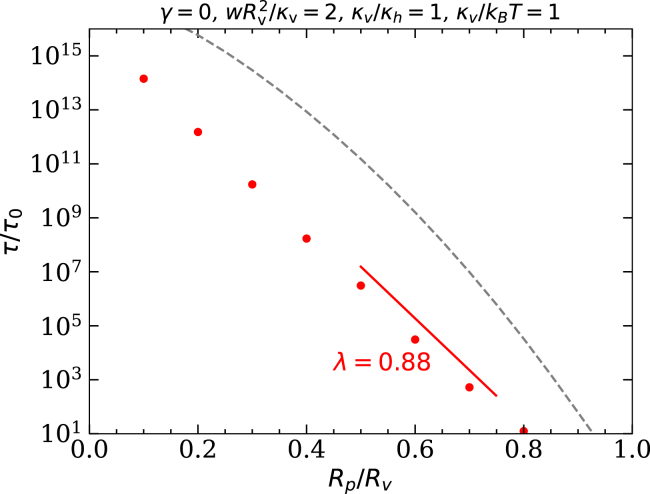
Translocation times at the pore-passage transitions for initially spherical vesicles with $R_{p}/R_{v}=0.5$, $\kappa_{v}/\kappa_{h}=1$, $\kappa_{h}/k_{B}T=1$, and γ=0 as a function of the pore-to-vesicle size ratio $R_{p}/R_{v}$, calculated using Eq. 3. Data are is shown for the spherical-cap model (*dashed line*) and triangulated membranes (*points*). The guide to the eye shows the characteristic exponential decay exponent λ in Eq. 4.

Here, E(ρ) refers to the difference of the total energy of the system calculated using Eq. 1 at translocation fractions $\rho_{1}$ and $\rho_{2}$, with the total energy in the free state, and $k_{0}$ is a measure for the friction between the vesicle and the pore. Fig. 5 shows the reduced translocation times $\tau /\tau_{0}$ for initially spherical vesicles translocating through pores of various radii, where $\tau_{0}=1/k_{0}$.

With the assumption of harmonic shapes for potential well and barrier, Eq. 3 can be reduced to the well-known Kramer’s escape problem (39). In our case, the exponent is determined by the height of the energy barrier,

(4) $$\tau \propto \exp\left[-\lambda \frac{R_{p}}{R_{v}}\frac{8\pi \left(\kappa_{v}+\kappa_{h}\right)}{k_{B}T}\right].$$

We expect the adjustment factor *λ* to be of the order 1. For fixed adhesion strength, the translocation times decrease approximately exponentially with increasing pore-to-vesicle size ratio $R_{p}/R_{v}$ (see Fig. 5). The exponential dependence of the translocation time on $R_{p}/R_{v}$ is expected from Eq. 4 because the barrier height $E_{\text{barrier}}\approx \left(1-\lambda R_{p}/R_{v}\right)8\pi \left(\kappa_{v}+\kappa_{h}\right)$ decreases almost linearly with increasing pore-to-vesicle size ratio (see Fig. 4 *a*). Thus, the translocation times show a high sensitivity to $R_{p}/R_{v}$. The deviation from the exponential decay accounts for the nonlinear dependence of the energy barrier on $R_{p}/R_{v}$ (see Fig. 4 *a*) and the exact shape of the energy landscape. In physiological conditions, we expect long translocation times because typical energy barriers are orders of magnitude higher than thermal energies. Furthermore, we find much shorter translocation times using triangulated membranes instead of the spherical-cap model, which reflects the lower energy barriers that we find for triangulated membranes and shows the importance of calculating accurate vesicle shapes.

## Translocation of initially prolate vesicles

**Figure 6:**
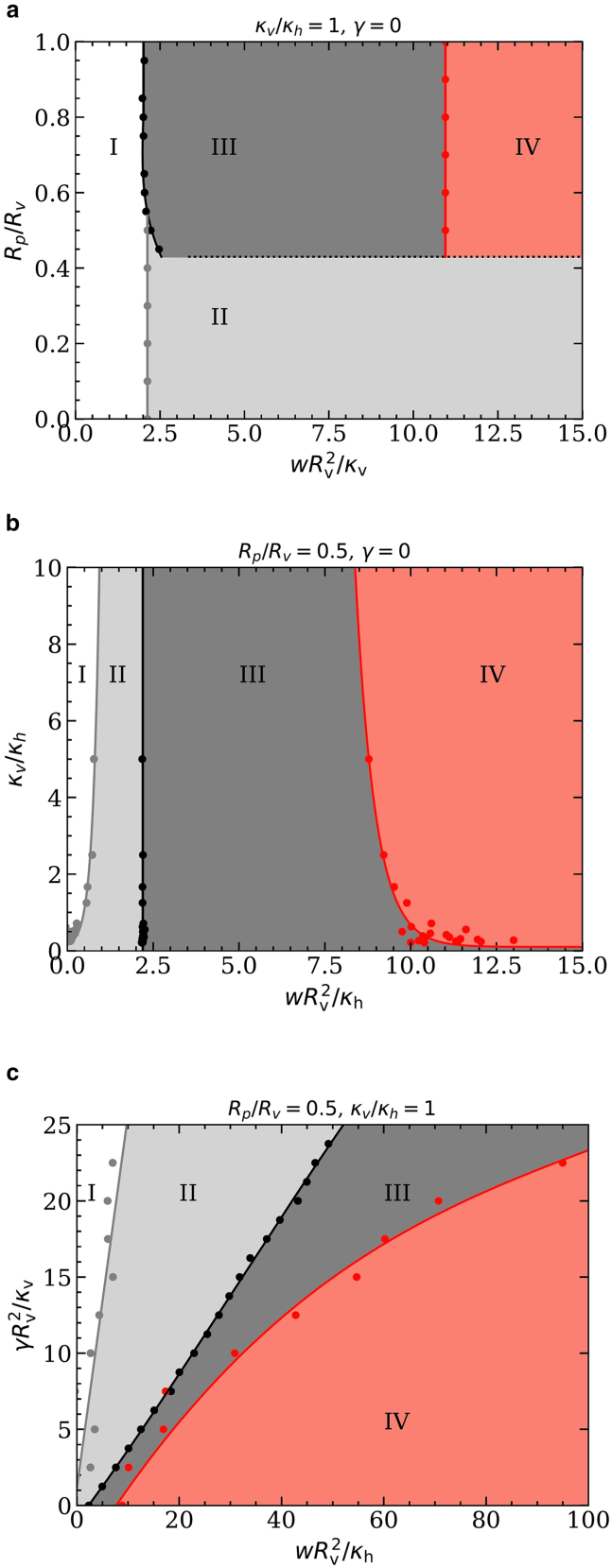
Translocation-state diagrams for prolate vesicles of v=0.8, (*a*) fixed $\kappa_{v}/\kappa_{h}=1$, various size ratios $R_{p}/R_{v}$ and adhesion strengths $wR_{v}^{2}/\kappa_{v}$; (*b*) fixed $R_{p}/R_{v}$, various bending-rigidity ratios $\kappa_{v}/\kappa_{h}$ and adhesion strengths $wR_{v}^{2}/\kappa_{h}$; (*c*) fixed $R_{p}/R_{v}$, $\kappa_{v}/\kappa_{h}=1$, various membrane tensions $\gamma R_{v}^{2}/\kappa_{v}$ and adhesion strengths $wR_{v}^{2}/\kappa_{v}$. The states are labeled following Fig. 3.

The shapes of prolate vesicles that have a constant reduced volume, translocating through pores with radii comparable to the lengths of the vesicles’ short axes, are only weakly affected by the constriction. Thus, the energy barriers for prolate vesicles are smaller than those for initially spherical vesicles with the same membrane area (compare Fig. 2). However, complete translocation of prolate vesicles does not occur if the enclosed vesicle volume of half-translocated vesicles cannot be accommodated by the available vesicle membrane area (see Fig. 6 *a*). Furthermore, for vesicles with reduced volume v=0.8, the range of adhesion strengths for stable deep-translocated states is considerably larger compared with the case of initially spherical vesicles. In this section, we discuss energy barriers and translocation times for cylindrically symmetric prolate-vesicle systems with reduced vesicle volumes v=0.8 and v=0.6; note that for reduced volume v=0.6 and spontaneous membrane curvature $c_{0}=0$ the prolate shape is metastable (40).

### Stable translocation states

The translocation is qualitatively different for prolate vesicles with reduced volumes v=0.8 and 0.6 compared with initially spherical vesicles. In particular, for v=0.8 and $R_{p}/R_{v}≲0.45$, complete translocation is inhibited because the vesicle’s membrane area is too small to enclose the volume for half-translocated states (compare Figs. 6 *a* and 3 *a*). For v=0.6, the weakly translocated state is suppressed for many parameter values because of the high bending-energy costs at the highly curved vesicle tip (see supporting material).

The adhesion strength for the binding transition is independent of the pore-to-vesicle size ratio but higher compared with initially spherical vesicles because the tip of the vesicle is wrapped first (compare Figs. 6 *a* and 3 *a*). However, for $R_{p}/R_{v}≲0.5$ and γ=0, the transition for prolate vesicles is continuous between the free and a shallow-translocated state. For $R_{p}/R_{v}≳0.5$, the transition is discontinuous and occurs directly between the free and a deep-translocated state. Because the vesicle locally flattens upon binding to the host membrane, the adhesion strength for the transition decreases with decreasing $\kappa_{v}/\kappa_{h}$ (see Fig. 6 *b*). Interestingly, for finite tension γ, the adhesion strength for the binding transition increases with increasing tension (see Fig. 6 *c*).

Fig. 6*a* and *b* show that the adhesion strength for the pore-passage transition depends only weakly on $R_{p}/R_{v}$ and $\kappa_{v}/\kappa_{h}$, as for initially spherical vesicles. However, for small values of $R_{p}/R_{v}$, the pore-passage transition is inhibited. With increasing host-membrane tension, the adhesion strength for the pore-passage transition increases. Because of the increased importance of the tension energy compared with the bending energy and the vesicle membrane area being identical in both cases, the adhesion strengths for the pore-passage transition are similar for the prolate and the initially spherical vesicles (compare Figs. 6 *c* and 3 *c*).

Analogously to the suppression of stable weak-translocated states by the prolate-vesicle shape, the stability of deep-translocated states is enhanced by the high bending-energy costs for wrapping the second tip of the vesicle; compare—in particular—Figs. 6 *a* and *b* and 3 *a* and *b*, where the effect is most pronounced. The transitions remain continuous for vanishing host-membrane tension, and the envelopment transition shifts to higher adhesion strengths upon decreasing $\kappa_{v}/\kappa_{h}$ (see Fig. 6 *b*). For $\kappa_{v}/\kappa_{h}\to 0$, the high deformation-energy cost for wrapping the highly curved tip of the vesicle to complete the translocation of a deep-translocated vesicle shifts the envelopment transition to $wR_{v}^{2}/\kappa_{v}\approx 15$, compared with $wR_{v}^{2}/\kappa_{v}\approx 4$ for initially spherical vesicles. With increasing host-membrane tension, the adhesion strength for the envelopment transition increases with increasing host-membrane tension (see Fig. 6 *c*). Unlike initially spherical vesicles, we find stable deep-translocated states for all host-membrane tensions.

### Energy barriers

**Figure 7:**
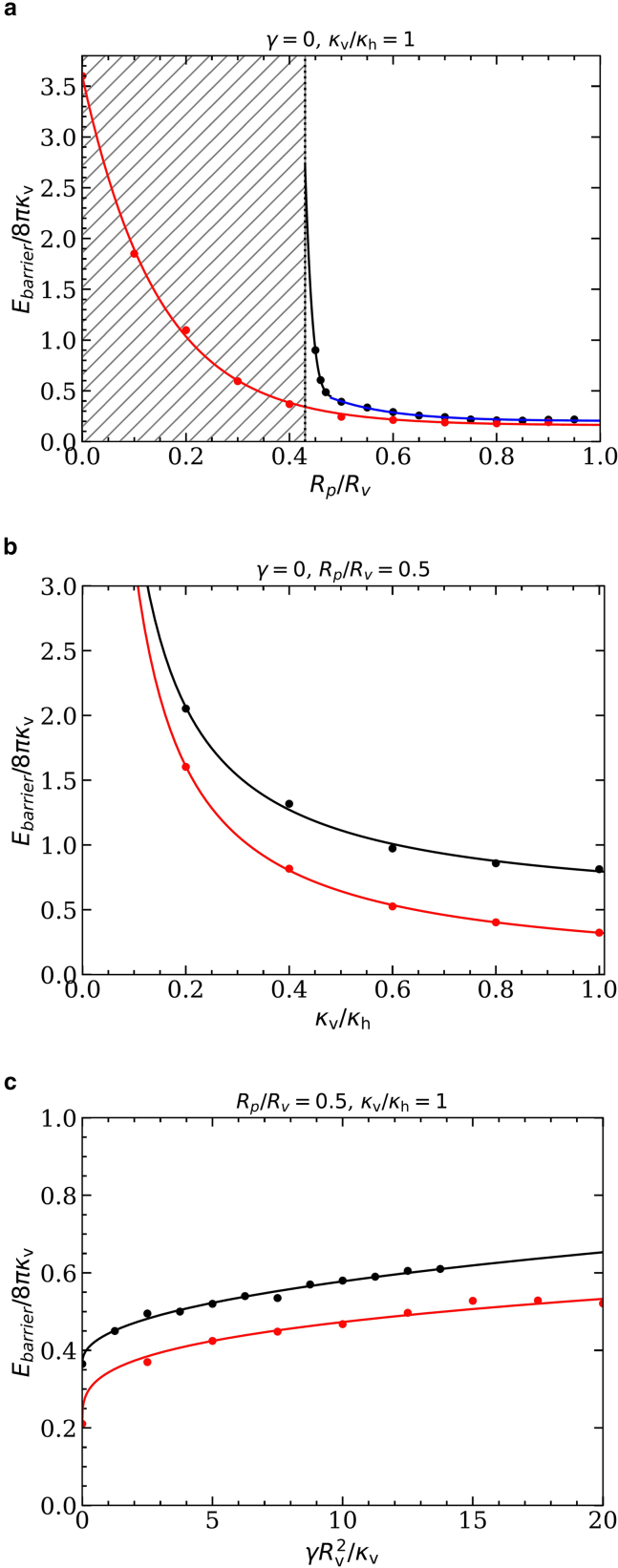
Energy barriers for the pore-passage transition of initially prolate vesicles with reduced volumes *v* = 0.6 (*red*) and v=0.8 (black/blue), and (*a*) $\kappa_{v}/\kappa_{h}=1$ and γ=0 as a function of $R_{p}/R_{v}$, (*b*) $R_{p}/R_{v}=0.5$ and γ=0 as a function of $\kappa_{v}/\kappa_{h}$, and (*c*) $R_{p}/R_{v}=0.5$ and $\kappa_{v}/\kappa_{h}=1$ as a function of membrane tension $\gamma R_{v}^{2}/\kappa_{v}$. In (*a*), the inaccessible parameter space for v=0.8 is marked by the shaded region; the blue line indicates the barrier originating from wrapping the vesicle tip, and the black line the barrier originating from the pore constriction; see supporting material. The fit functions for the energy barriers are provided in the supporting material.

The energy barriers for the translocation of initially prolate vesicles with reduced volumes v=0.8 and v=0.6 through pores with fixed radii are shown in Fig. 7 *a*–*c* with respect to the pore-to-vesicle size ratio, the bending-rigidity ratio of vesicle and host membranes, and the host-membrane tension, respectively.

For pore-to-vesicle size ratios $R_{p}/R_{v}≳0.6$, the energy barrier for the pore-passage transition remains approximately constant because the vesicles are weakly or not at all constricted by the pore (see Fig. 7 *a*). The barrier originates from the high bending-energy costs for wrapping the tip of the vesicle and, therefore, does not vanish for $R_{p}/R_{v}\to 1$. For $R_{p}/R_{v}≲0.6$, the dependence of the barrier height on the pore-to-vesicle size ratio is qualitatively different for the two reduced vesicle volumes. Although for v=0.8 the energy barrier diverges at $R_{p}/R_{v}\approx 0.44$ where the vesicle membrane area is too small to allow the vesicle to squeeze through the pore, for v=0.6 the barrier increases smoothly with decreasing $R_{p}/R_{v}$ until an infinitesimally small pore size. The threshold reduced volume above that the energy diverges can be analytically calculated by equating the total area for two equal-sized spherical caps connected to the pore to the vesicle membrane area $4\pi R_{v}^{2}$, which results in

(5) $$v=\frac{\sqrt{2-{\left(R_{p}/R_{v}\right)}^{2}}\left({1+\left(R_{p}/R_{v}\right)}^{2}\right)}{2}\text{.}$$

For $R_{p}/R_{v}=0.5$ and various bending-rigidity ratios $\kappa_{v}/\kappa_{h}$, the contribution of the host membrane dominates the energy barrier for small values of $\kappa_{v}/\kappa_{h}$ but has a negligible contribution for high values of $\kappa_{v}/\kappa_{h}$ (see Fig. 7 *b*). For sufficiently high ratios $\kappa_{v}/\kappa_{h}$ and v=0.8, the energy barrier increases linearly with $\kappa_{v}$, whereas for v=0.6 the energy barrier vanishes (not shown). In the latter case, the vesicle translocates without being constricted by the pore, and the characteristic energy for deforming the host membrane is small compared with the characteristic energy for deforming the vesicle.

For $R_{p}/R_{v}=0.5$ and very small host-membrane tensions, $\gamma \ll \kappa_{v}/R_{v}^{2}$, the energy barrier initially increases strongly with increasing γ (see Fig. 7 *c*). For large tensions, $\gamma >\kappa_{v}/R_{v}^{2}$, the energy barrier increases weakly with increasing host-membrane tension. The dependence of the barrier height on the host-membrane tension is qualitatively similar for both reduced volumes, but the absolute barrier height is lower for prolate vesicles with v=0.6 compared to prolate vesicles with v=0.8.

### Translocation times

**Figure 8:**
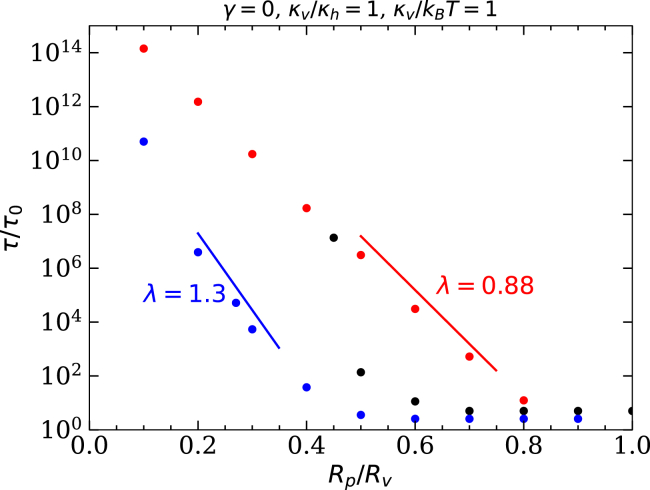
Translocation times at the pore-passage transitions for initially spherical vesicles with $\kappa_{v}/\kappa_{h}=1$, $\kappa_{h}/k_{B}T=1$, and γ=0 as a function of the pore-to-vesicle size ratio $R_{p}/R_{v}$, calculated using Eq. 3. Data are shown for an initially spherical vesicle (*red*), a prolate vesicle with v=0.8 (*black*), and a prolate vesicle with v=0.6 (*blue*). The guides to the eye show the characteristic decay exponents λ in Eq. 4.

The translocation times calculated using Eq. 3 show an exponential decrease with increasing pore-to-vesicle size ratio $R_{p}/R_{v}$ (see Fig. 8). The decay constant is smaller for the initially spherical than for the prolate vesicles with v=0.6, and typical translocation times are orders of magnitude longer for the initially spherical vesicles. In the case of the diverging energy barrier for v=0.8, the translocation time also diverges. For high values of $R_{p}/R_{v}$, the translocation times for the prolate vesicles are independent of $R_{p}/R_{v}$, as are the energy barriers (see Fig. 7 *a*). However, the characteristic times for overcoming the wrapping-energy barrier for the tips are significantly smaller than those for the barriers originating from the pores constricting the vesicles.

## Discussion and conclusions

In experiments, vesicle and cell shapes can often be quantified well using optical microscopy, but forces cannot be measured as easily. Computer simulations help us to connect vesicle shapes with energies and forces. Using a continuum-membrane model and exploiting cylindrical symmetry, we have studied the translocation of initially spherical and prolate vesicles through circular pores with fixed radii. Vesicle translocation is driven by an adhesion-energy gain through a contact interaction with a pore-spanning host membrane, which at the same time contributes to the deformation-energy costs. The driving mechanism for the translocation by adhesion and wrapping differs from an osmotic-pressure difference on both sides of the pore studied previously (20,21,22,24). Our predictions for the dependence of the energy barrier on the pore size and the phase behavior on the membrane bending rigidities can be tested experimentally in model systems. Therefore, our results may help to understand cellular uptake processes, such as passive endocytosis of extracellular vesicles, budding of virions in the presence of a cortical host cytoskeleton, and invasion of parasites into host cells.

We find an exponential decay of the translocation times with increasing pore-to-vesicle size ratios. The deformation-energy landscapes have their maxima before half translocation because of the energy required to deform the host membrane. Significant translocation rates driven by thermal motion are expected for $\kappa_{v}\approx \kappa_{h}\approx k_{B}T$. However, energy barriers comparable to thermal energies are known for entropy-dominated systems, such as pore-passage of linear polymer chains (41,42,43,44,45). The barrier heights for vesicle-pore translocation are determined by the curvature-elastic properties of the fluid membranes, and physiological lipid-bilayer bending rigidities are of the order of $\kappa =50\;k_{B}T$ (46). Therefore, to experimentally observe thermal translocation, a sufficiently high vesicle-host-membrane adhesion strength is required that reduces the activation energy.

We have studied vesicles with reduced volumes similar to v=0.84 for *Plasmodium falciparum* merozoites (12) and v=0.57 for *Toxoplasma gondii* tachyzoites (47); our vesicle model goes beyond the established approach to simulate *Plasmodium falciparum* merozoites as hard egg-shaped particles (12). Using membrane bending rigidities $\kappa_{v}=\kappa_{h}=50\;k_{B}T$ and a biologically relevant tension $\gamma =0.003\;\mathrm{dyn}\;cm^{-1}=750\;k_{B}T\;\mu m^{-2}$ for the host membrane (48), we estimate that a stable complete-translocated state of *Plasmodium* requires an adhesion strength $w\approx 10^{4}\;k_{B}T\;\mu m^{-2}$, which can be achieved by receptor-ligand bonds (12,49). Our finding of suppressed complete wrapping with increasing host-membrane tension has also been reported for the invasion of *Plasmodium* in erythrocytes (50). However, our vesicle model differs in many respects from actual parasites, not including the parasite’s nucleus (14), neglecting slow membrane tension propagation (51), and lacking cytoskeletal filaments. In particular, the subpellicular microtubules that are anchored to the parasite’s apical complex, filamentous actin, and a potential bulk elasticity of the parasites may significantly affect free-parasite shapes and deformabilities; an actin ring may form the “pore.”

For *Toxoplasma*, the microtubule cytoskeleton remains intact during invasion, reducing parasite deformability compared to a membrane-only model. However, recent microscopy studies show that *Plasmodium* merozoites contain only two to three very short subpellicular microtubules (52) and experience major shape deformations when squeezing through the tight junction (15,53). This supports the use of a vesicle model for invasion; the high physiological osmotic concentration justifies assuming a fixed parasite reduced volume in our calculations. The deformability of vesicle-like parasites may also explain why—contrary to the widely accepted notion of invasion occurring from the merozoite’s pointed end—recent experiments hint that invasion for *Plasmodium knowlesi* starts from the flattened end (54,55).

Our cell-scale calculations focus on parasite translocation across the tight junction. Modeling further aspects of parasite-host invasion requires additional components of the model or even additional simulation techniques. A prominent example is parasite reorientation, such that its apical end points toward the host’s plasma membrane. For example, a receptor gradient on the parasite surface with a higher adhesion strength at the apical compared with the dorsal end (12) and also an interplay of red-blood-cell deformability and receptor-ligand bond dynamics (56,57) have been hypothesized as potential mechanisms and quantified using simulations. Similarly, the detachment of the parasitophorous vacuole from the host plasma membrane after translocation requires a detailed study of neck fission (58). Thus, complete pore translocation does not yet guarantee successful host-cell invasion but is a necessary prerequisite.

Experimentally reported in vitro invasion times for *Plasmodium falciparum* merozoites (59,60) and *Toxoplasma gondii* tachyzoites (14,61) are ≈15s. The invasion time of *Toxoplasma* with blocked myosin A in nonprofessional phagocytes—and thus without active parasite motor forces—has been reported to be ≈120s (62). The first-passage times we calculate using the Fokker-Planck approach are obtained from the barrier heights and shapes. Our results show that the times are very sensitive to both pore size and initial vesicle shape. However, Eq. 3 cannot provide absolute values without knowing the friction coefficient $k_{0}$ for translocation. Moreover, our calculations are based on minimal-energy landscapes and do not consider vesicle and host-membrane shape fluctuations. Therefore, and because neither active motor forces for parasite invasion nor the kinetics of receptor-ligand binding are taken into account, our model cannot be expected to quantitatively predict the dynamics of parasite invasion.

Our model of vesicles and fixed pores may be considered as a biophysical approach for studying host invasion of apicomplexans that captures parasite and host-membrane bending rigidities and tensions, the prolate shape of free parasites, and parasite deformability. However, we may miss crucial mechanical aspects by assuming a pore with a fixed radius instead of an elastic pore and not including parasite and host cytoskeletal filaments. We plan to add further mechanical building blocks for modeling host entry of *Plasmodium* merozoites and *Toxoplasma* tachyzoites in the future, as well as active motor forces that drive parasite invasion.

## Acknowledgments

N.B., A.K.D., and T.A. acknowledge funding by the 10.13039/501100001659Priority Program SPP 2332 “Physics of Parasitism” of Deutsche Forschungsgemeinschaft (DFG), and helpful discussions with Mirko Singer (Heidelberg), and Javier Periz and Markus Meissner (Munich).

## Author contributions

A.K.D., G.G., and T.A. conceived the project. A.K.D. and T.A. designed the research. N.B. and J.M. developed the code. N.B. performed the numerical calculations and analyzed the data. All authors discussed the data and wrote the manuscript.

## Declaration of interests

The authors declare no competing interests.

## Appendix A: Spherical-cap model

The energetics for an initially spherical vesicle translocating through a pore can be estimated using the spherical-cap model, where the fractions of the vesicle membrane on both sides of the pore are represented as spherical caps. Assuming vanishing spontaneous curvature, the integrals in Eq. 1 can be solved analytically,

(6) $$\begin{array}{ccc} E & = & 2\kappa_{v}\left(S_{v}\rho \;\frac{1}{R_{1}^{2}}+S_{v}\left(1-\rho \right)\;\frac{1}{R_{2}^{2}}\right)+2\kappa_{p}\;S_{v}\rho \;\frac{1}{R_{1}^{2}}\\ & & +\gamma \;\left(S_{v}\rho -\pi R_{p}^{2}\right)-w\;S_{v}\rho \text{.} \end{array}$$

Here,

(7) $$\begin{array}{ccc} R_{1} & = & \frac{S_{v}\rho }{\sqrt{4\pi S_{v}\rho -4\pi^{2}R_{p}^{2}}} \end{array}$$

and

(8) $$R_{2}=\frac{S_{v}\left(1-\rho \right)}{\sqrt{4\pi S_{v}\left(1-\rho \right)-4\pi^{2}R_{p}^{2}}}$$

are the radii of the spherical caps for the membrane that has and has not translocated through the pore, respectively.

Eq. 6 is applicable when the vesicle touches the rim of the pore. When the vesicle does not touch the rim, in the spherical-cap model, it remains spherical with radius $R_{v}$, and the total energy is the energy for wrapping a spherical particle,

(9) $$\begin{array}{ccc} E & = & 2\kappa_{v}\;S_{v}\frac{1}{R_{v}^{2}}+2\kappa_{p}\;S_{v}\rho \;\frac{1}{R_{v}^{2}}\\ & & +\gamma \;\left(S_{v}\rho -\pi r_{\text{cl}}^{2}\right)-w\;S_{v}\rho \text{,} \end{array}$$

where $r_{\text{cl}}<R_{p}$ is the radius of the contact line,

(10) $$r_{\text{cl}}=\sqrt{R_{v}^{2}-{\left(R_{v}-2R_{v}\rho \right)}^{2}}\text{.}$$

## Appendix B: Energy minimization

The membrane deformation energies are calculated using the named quantity “star_perp_sq_mean_curvature” in Surface Evolver (32), where the bending energy is discretized over the vertices. The total bending energy is calculated as a sum over all membrane vertices besides those on the circular wireframe bounding the system, and the tension energy as a sum over all facets. In continuum models, the existence of metastable states is common; see, e.g., Fig. 2 and Refs. (33,34,63). Assuming prolate-vesicle shapes that resemble the parasite shapes during vesicle-pore translocation, we find the lowest-energy states to report as stable states by starting the minimization using various initial system configurations.

Membrane bending rigidities are assigned separately to the vesicle and the pore-spanning membranes. The pore is modeled as a circular constraint independent of the contact line where the pore-spanning membrane detaches from the vesicle (but is found to coincide). Named quantities for the areas of the translocated and not-translocated vesicle membrane allow us to calculate deformation energies for arbitrary wrapping fractions.

Exploiting the spherical symmetry of the system, we simulate $1/16^{\text{th}}$ of the system. Initially, two facets are defined for the free vesicle membrane, two for the bound vesicle and host membranes, and two for the free host membrane. To minimize the deformation energy, gradient-descent and conjugate-gradient minimization steps were used alternatingly with refinements of the discretization of the membrane to achieve both efficient minimization and the desired accuracy. Equiangulation and vertex averaging are regularly performed to maintain approximately equilateral triangles with areas similar to those of their neighboring triangles; special care needs to be taken for equal triangle sizes across the contact line where the host membrane detaches from the vesicle. Toward the end of the minimization for a specific refinement level, we use the “hessian_seek” command to ensure we are close to the energy minimum.

## Supporting material

Document S1. Figures S1–S12

Document S2. Article plus supporting material
