# Supplementary material for: Adhesion-driven vesicle translocation through membrane-covered pores
Source: Biophys J. 2025 Jan 24;124(5):740–52. doi: 10.1016/j.bpj.2025.01.012 (PMC11897550; doi:10.1016/j.bpj.2025.01.012)
Supplement: Document S1. Figures S1–S12 [file mmc1.pdf]

Biophysical Journal, Volume *124*

Supplemental information

Adhesion-driven vesicle translocation through membrane-covered pores

Nishant Baruah, Jiarul Midya, Gerhard Gompper, Anil Kumar Dasanna, and Thorsten Auth

Supporting Information: Adhesion-driven vesicle translocation through membrane-covered pores

Nishant Baruah,[1] Jiarul Midya,[1,2] Gerhard Gompper,[1] Anil Kumar Dasanna,[3,4,5] and Thorsten Auth[1]

[1] Theoretical Physics of Living Matter, Institute of Biological Information Processing and Institute for Advanced Simulation, Forschungszentrum Jülich, 52425 Jülich, Germany
[2] School of Basic Sciences, Indian Institute of Technology, Bhubaneswar, 752050, India
[3] Department of Theoretical Physics and Center for Biophysics, Saarland University, Saarbrücken, Germany
[4] INM–Leibniz Institute for New Materials, Campus D2 2, 66123 Saarbrücken, Germany
[5] Department of Physical Sciences, Indian Institute of Science Education and Research Mohali, Sector 81, Knowledge City, S. A. S. Nagar, Manauli PO 140306, India

S1. SPHERICAL-CAP MODEL

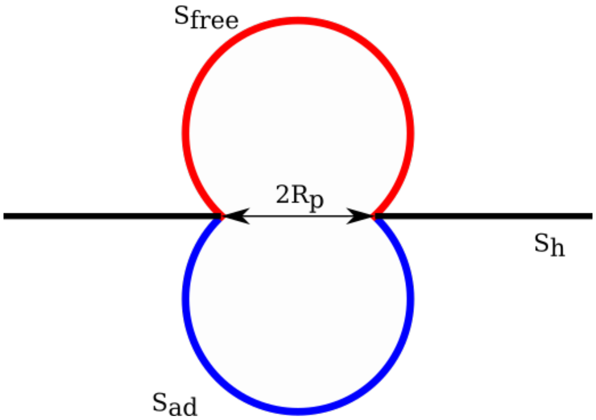

FIG. S1: Shape of an initially spherical vesicle of radius R_v translocating through a circular pore of radius $R_\mathrm{p} = R_\mathrm{v}/2$ in a host membrane with bending rigidity κ_h and tension $\gamma R_\mathrm{v}^2/\kappa_\mathrm{h} = 0$, simulated using the spherical-cap model. The snapshot corresponds to the vesicle-membrane translocation fraction $\rho = 0.5$; S_free, S_ad and S_h indicate the free vesicle membrane, the double-bilayer of the vesicle membrane adhered to the host membrane that is bounded by the pore, and the free host membrane, respectively.

Figure S1 shows a vesicle shape obtained using the spherical-cap model, see App. A. Partial-translocated vesicles are constructed by two spherical caps, ensuring that the total vesicle area $S_\mathrm{v} = S_\mathrm{free} + S_\mathrm{ad}$ remains constant during translocation. Comparing the spherical-cap shape to the shape obtained using triangulated membranes, see Fig. 1, it becomes obvious that the major difference is the shape of the neck region formed near the pore during translocation.

Figure S2 shows the energy landscapes of initially spherical vesicles translocating through a pore of radius $R_\mathrm{p} = R_\mathrm{v}/2$ calculated by spherical-cap and triangulated-membrane models at the pore-passage transition (w=2). As discussed in Sec. III B, the energy barriers predicted using triangulated-membranes are lower because of the higher number of degrees of freedom which yield vesicle shapes more favorable for translocation, as compared to spherical-caps.

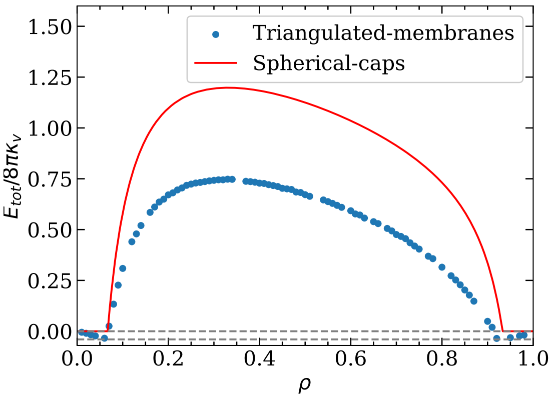

FIG. S2: Energies as a function of translocation fraction ρ of an initially spherical vesicle translocating through a pore of radius $R_\mathrm{p} = R_\mathrm{v}/2$ at the pore-passage transition, $\tilde{w} = 2$; calculated using spherical-cap and triangulated-membrane models.

S2. ENERGY-BARRIER HEIGHTS OBTAINED USING THE SPHERICAL-CAP MODEL

In Fig. 4(a), the height of the energy barrier at the pore-passage transition, estimated using the spherical-cap model, is fit by a negative exponentially growing function $E_\mathrm{barrier}/8\pi\kappa_\mathrm{v} = 3.55 - 2.27^{(R_\mathrm{p}/R_\mathrm{v}+0.54)}$.

In Fig. 4(b), the height of the energy barrier is fit by the decaying function $E_\mathrm{barrier}/8\pi\kappa_\mathrm{v} = 0.75 + 0.44/(\kappa_\mathrm{v}/\kappa_\mathrm{h})$.

In Fig. 4(c), the height of the energy barrier is fit by a constant $E_\mathrm{barrier}/8\pi\kappa_\mathrm{v} = 1.25$.

S3. ENERGY-BARRIER HEIGHTS OBTAINED USING TRIANGULATED MEMBRANES

In Fig. 4(a), the height of the energy barrier calculated using triangulated membranes is fit by $E_\mathrm{barrier}/8\pi\kappa_\mathrm{v} = -1.89 + 0.48^{(R_\mathrm{p}/R_\mathrm{v}-1.84)}$.

In Fig. 4(b), the triangulated-membrane data is fit by $E_\mathrm{barrier}/8\pi\kappa_\mathrm{v} = 0.38 + 0.40/(\kappa_\mathrm{v}/\kappa_\mathrm{h})$.

In Fig. 4(c), the triangulated-membrane data is fit by a power law $E_\mathrm{barrier}/8\pi\kappa_\mathrm{v} = 0.78 + 0.04(\gamma R_\mathrm{v}^2/\kappa_\mathrm{v})^{0.47}$.

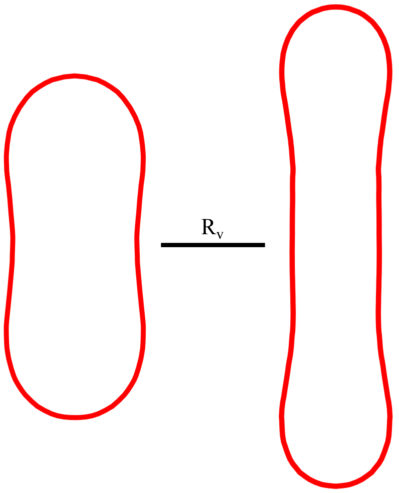

FIG. S3: Cylindrically symmetric shapes of free prolate vesicles with $v = 0.8$ (left) and 0.6 (right). The scale bar represents the radius of a spherical vesicle with the same surface area S_v.

In Fig. 7(a), the data for $v = 0.6$ is fit by an exponentially decaying function $E_\mathrm{barrier}/8\pi\kappa_\mathrm{v} = 0.2 + 0.0004^{(R_\mathrm{p}/R_\mathrm{v}-0.17)}$. The data for $v = 0.8$ vesicle for large $R_\mathrm{p}/R_\mathrm{v}$ is fit by an exponentially decaying function $E_\mathrm{barrier}/8\pi\kappa_\mathrm{v} = 0.2 + 0.00001^{(R_\mathrm{p}/R_\mathrm{v}-0.31)}$, and for small $R_\mathrm{p}/R_\mathrm{v}$ by a normal distribution function with extremely small standard deviation σ, similar to the definition of the Dirac delta function, $E_\mathrm{barrier}/8\pi\kappa_\mathrm{v} = e^{((R_\mathrm{p}/R_\mathrm{v}-0.38)/0.04)^2}/(|0.04|\sqrt{\pi}) + 0.43$.

In Fig. 7(b), the data for $v = 0.8$ is fit by $E_\mathrm{barrier}/8\pi\kappa = 0.48 + 0.32/(\kappa_\mathrm{v}/\kappa_\mathrm{h})$. The data for $v = 0.6$ is fit by $E_\mathrm{barrier}/8\pi\kappa = 0.002 + 0.32/(\kappa_\mathrm{v}/\kappa_\mathrm{h})$

In Fig. 7(c), the data for $v = 0.6$ and $v = 0.8$ is fit by the power law $E_\mathrm{barrier}/8\pi\kappa = 0.28 + 0.16(\gamma R_\mathrm{v}^2/\kappa_\mathrm{v})^{0.42}$ and $E_\mathrm{barrier}/8\pi\kappa = 0.21 + 0.15(\gamma R_\mathrm{v}^2/\kappa_\mathrm{v})^{0.26}$, respectively.

S4. FREE PROLATE VESICLES

Prolate free-vesicle shapes are locally stable for all reduced volumes $v < 1$ [1]. Figure S3 shows the equilibrium shapes of prolate vesicles with reduced volumes $v = 0.6$ and 0.8.

S5. ENERGIES FOR PROLATE-VESICLE SYSTEMS DURING PORE-PASSAGE

The energy landscapes of prolate vesicles with reduced volumes $v = 0.8$ and 0.6 at their pore-passage transitions are shown in Fig. S4. Comparing them with those for initially spherical vesicles in Fig. S2, note the lower barrier heights and the more complex shapes of the barriers for the prolate vesicles.

Figure S5 shows the energy barriers for prolate vesicles with $v = 0.8$ and $R_\mathrm{p}/R_\mathrm{v} = 0.45$, 0.47, and 0.48. There are two peaks for the translocation-energy barrier: the first, $\Delta\tilde{E}_{b1}$, is due to wrapping the tip of the prolate vesicle translocating in rocket orientation, and the second, $\Delta\tilde{E}_{b2}$, is due to the constriction of the vesicle by the pore. For prolate vesicles, which are thinner than initially spherical vesicles, $\Delta\tilde{E}_{b1}$ dominates for most pore sizes. However, for a sufficiently narrow pore, $\Delta\tilde{E}_{b2}$ dominates. Here, the energy eventually diverges when the fixed vesicle membrane area is too small to allow the vesicle with fixed volume to squeeze through the pore. This crossover, from the domination of the first to the second peak, occurs around $R_\mathrm{p}/R_\mathrm{v} = 0.47$, see Fig. S5; $\Delta\tilde{E}_{b2}$ rises very sharply upon further decreasing $R_\mathrm{p}/R_\mathrm{v}$.

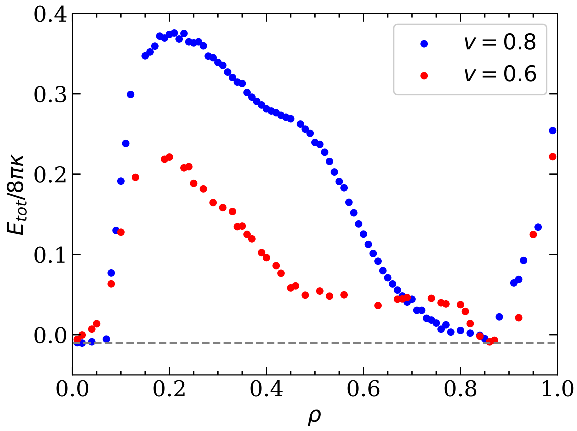

FIG. S4: Energy landscapes for prolate $v = 0.8$ and 0.6 vesicles, translocating through a pore of radius $R_\mathrm{p} = R_\mathrm{v}/2$ as a function of translocation fraction ρ, calculated at the pore-passage transitions for $\tilde{w} = 2.24$ and 3.6, respectively.

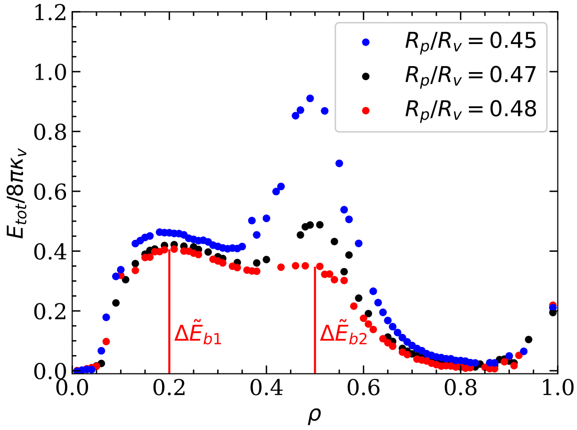

FIG. S5: Energies for a $v = 0.8$ vesicle at $w = 2.24$, showing the two energy barriers formed $\Delta\tilde{E}_{b1}$ and $\Delta\tilde{E}_{b2}$ and the dominance of $\Delta\tilde{E}_{b2}$ over $\Delta\tilde{E}_{b1}$ at $R_p/R_v < 0.48$. $\Delta\tilde{E}_{b2}$ increases sharply with a decrease in R_p/R_v until it diverges near $R_\mathrm{p}/R_\mathrm{v} = 0.435$.

S6. TRANSLOCATION FRACTION FOR SPECIAL POINTS

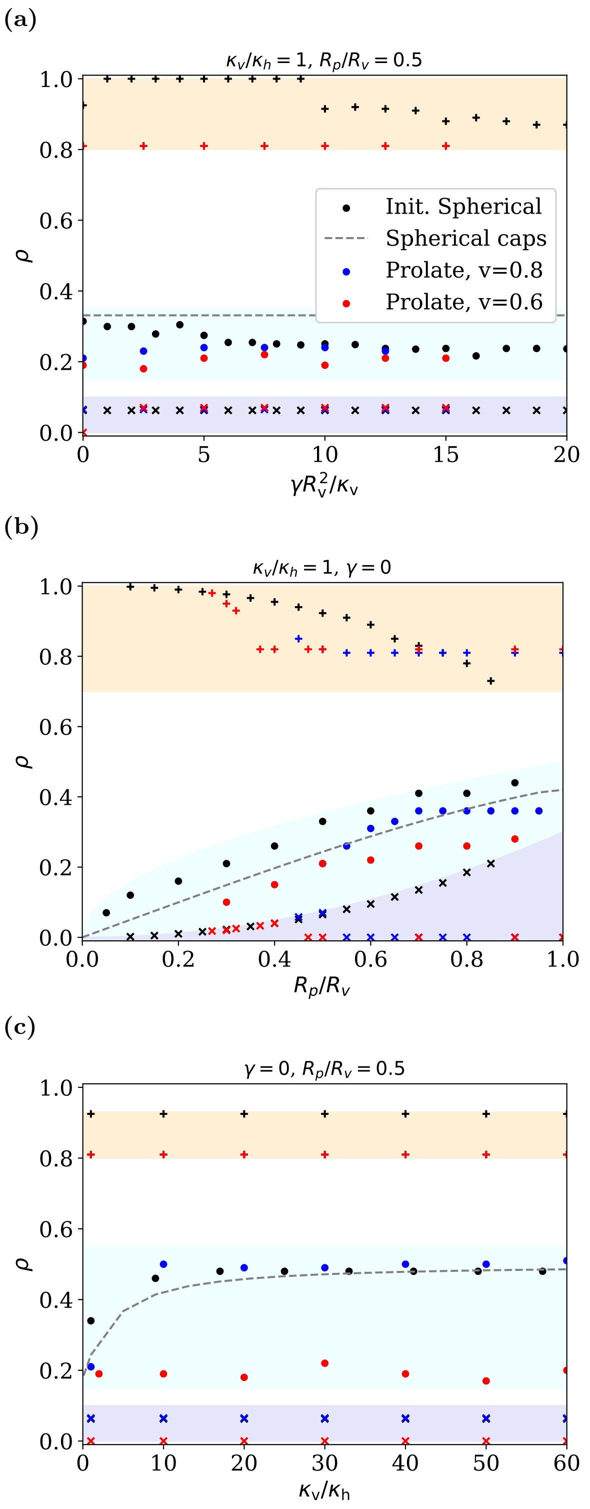

FIG. S6: Translocation fractions of special points in the energy landscape at the pore-passage transition, compare Fig. 4. The points in lavender mark shallow-translocated states, the points in orange stable deep-translocated states, and the points in blue energy-barrier maximums.

Figure S6 shows the translocation fractions ρ of the shallow- and deep-translocated states that coexist at the pore-passage transition, as well as the translocation fraction for the energy-barrier maximums for various host-membrane tensions, pore sizes, and vesicle-to-host membrane bending-rigidity ratios.

For various membrane tensions $\gamma R_\mathrm{v}^2/\kappa_\mathrm{v}$, shallow-translocated states around $\rho = 0.07$ coexist with deep- and complete-translocated states for initially spherical and prolate vesicles with $v = 0.8$ and 0.6. For $\gamma = 0$ and a $v = 0.6$ prolate vesicle, the non-translocated state at $\rho = 0$ coexists with a deep-translocated state. The translocation fractions for the deep-translocated states are around $\rho = 0.8$ for both prolate vesicles around $\rho = 0.8$; beyond that, the curved tips of the prolate vesicles have to be wrapped. For initially spherical vesicles beyond $\gamma R_\mathrm{v}^2/\kappa_\mathrm{v} = 1$ and up to $\gamma R_\mathrm{v}^2/\kappa_\mathrm{v} = 10$, the complete-translocated state at $\rho = 1$ coexists with a shallow-translocated state, see also Fig 3(c). The energy-barrier maximums for the prolate vesicles are located around $\rho = 0.2$, which corresponds to the wrapping of the first highly curved tip. The energy barriers for initially spherical vesicles are around $\rho = 0.35$, which is also predicted by the spherical-cap model. However, the location of the barrier shifts to lower values of ρ with increasing $\gamma R_\mathrm{v}^2/\kappa_\mathrm{v}$, where more energy is needed to deform the host membrane compared to the vesicle.

With increasing pore-to-vesicle size ratio $R_\mathrm{p}/R_\mathrm{v}$, the translocation fractions of the shallow-translocated states coexisting with complete- and deep-translocated states shift to higher ρ; this holds for all three systems that we studied. However, beyond a threshold pore-to-vesicle size ratio, the prolate vesicles pass through without deformation, and a direct transition from the non-translocated to the deep-translocated state is observed. Similarly, the translocation fractions for the coexisting deep-translocated states initially shift to lower values of ρ with increasing $R_\mathrm{p}/R_\mathrm{v}$ but then remain constant around $\rho = 0.8$, which corresponds to translocation fraction beyond that the second tip of the prolate vesicles gets wrapped. The location of the energy-barrier maximums shifts to higher values of ρ with increasing $R_\mathrm{p}/R_\mathrm{v}$, which is also shown by the spherical-cap model.

For various vesicle-to-host membrane bending rigidity ratios $\kappa_\mathrm{v}/\kappa_\mathrm{h}$ and $R_\mathrm{p}/R_\mathrm{v} = 0.5$, the coexisting shallow-translocated states for both the initially-spherical vesicle and the $v = 0.8$ prolate vesicle are at $\rho \approx 0.07$. For $v = 0.6$ prolate vesicles, the non-translocated state coexists with the deep-translocated state. The coexisting deep-translocated states have translocation fractions $\rho \approx 0.92$ for initially spherical vesicles and $\rho \approx 0.8$ for both prolate vesicles. The energy-barrier maximums for $v = 0.6$ prolate vesicles is at $\rho \approx 0.2$, which corresponds to the wrapping the first tip, compare Fig. S5. The translocation fractions for the energy-barrier maximums for $v = 0.8$ prolate vesicles, however, increase strongly with increasing $\kappa_\mathrm{v}/\kappa_\mathrm{h}$ from $\rho = 0.2$ to $\rho = 0.5$, which corresponds to the translocation fraction for the second

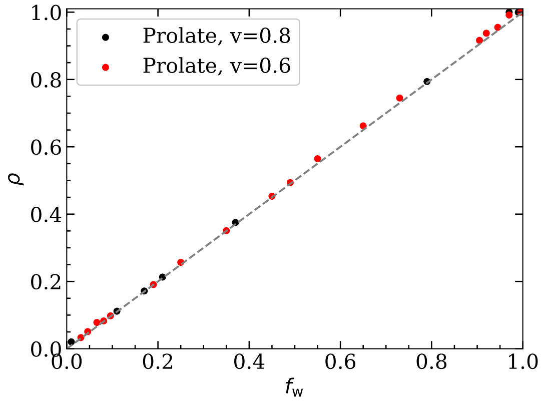

FIG. S7: Translocation fraction ρ as a function of wrapping fraction f_w for prolate vesicles. The dotted line $\rho = f_\mathrm{w}$ is a guide to the eye to detect where ρ deviates from f_w.

barrier, see Fig. S5. There is also an increase in ρ for the energy maximums for initially spherical vesicles calculated using the spherical-cap model; with increasing $\kappa_\mathrm{v}/\kappa_\mathrm{h}$ the deformation of the vesicle becomes more significant to the barrier created and the maximum of the energy barrier is at $\rho = 0.5$, as for the translocation of a vesicle through a pore without the presence of a host membrane.

S7. TRANSLOCATION VS. WRAPPING FRACTION

For adhesion-driven pore translocation, the contact line between the vesicle and the pore-spanning membrane and the pore itself do not have to coincide. Therefore, also the tranlocation fraction ρ and the wrapping fraction f_w of the vesicle membrane can be different. In Fig. S7, we compare translocation fraction and wrappping fraction f_w for the 'thin' prolate vesicles, for that a deviation between both quantities may appear most likely. However, we find that both quantities agree within our calculation accurracty for most values of f_w. We find small deviations for very small and large values of f_w, where the cylindrical radius of the vesicle is much smaller than the pore radius and thus $\rho > f_\mathrm{w}$; we have considered this for our calculations of energy landscapes, translocation diagrams, energy barriers, as well as translocation times for the initially spherical as well as both prolate versicles.

S8. STABLE TRANSLOCATION STATES FOR PROLATE $v = 0.6$ VESICLES

The translocation-state diagrams for prolate $v = 0.6$ vesicles and $R_\mathrm{p} = R_\mathrm{v}/2$ are similar to those for $v = 0.8$ vesicles, see Fig. 6. The major differences arise because the pore-passage transition for $v = 0.6$ vesicles is also the binding transition, whereas stable shallow-translocated

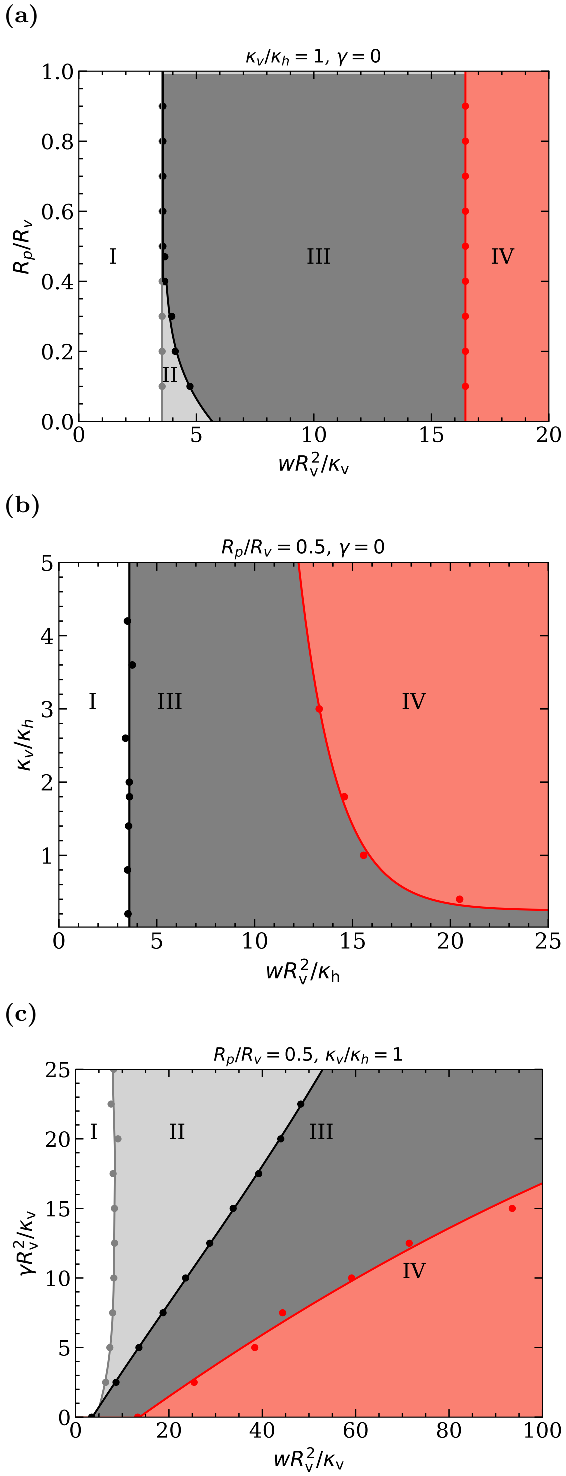

FIG. S8: Translocation-state diagrams for prolate $v = 0.6$ vesicles for (a) $\kappa_\mathrm{v}/\kappa_\mathrm{h} = 1$, $\gamma = 0$, and various size ratios $R_\mathrm{p}/R_\mathrm{v}$ and adhesion strengths $wR_\mathrm{v}^2/\kappa_\mathrm{v}$, (b) $R_\mathrm{p}/R_\mathrm{v} = 0.5$, $\gamma = 0$, and various bending-rigidity ratios $\kappa_\mathrm{v}/\kappa_\mathrm{h}$ and adhesion strengths $wR_\mathrm{v}^2/\kappa_\mathrm{h}$, and (c) $R_\mathrm{p}/R_\mathrm{v} = 0.5$, $\kappa_\mathrm{v}/\kappa_\mathrm{h} = 1$, and various host-membrane tensions $\gamma R_\mathrm{v}^2/\kappa_\mathrm{v}$ and adhesion strengths $wR_\mathrm{v}^2/\kappa_\mathrm{v}$.

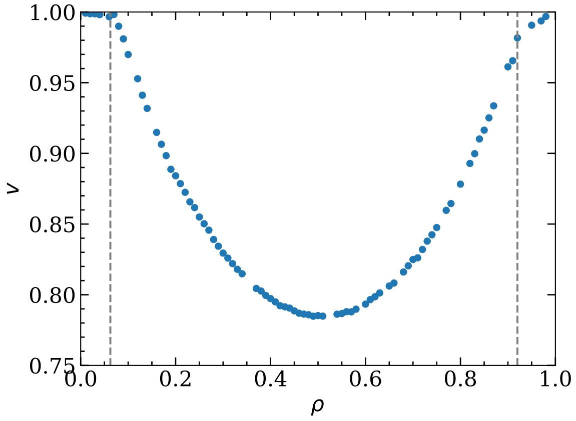

FIG. S9: Reduced volumes v of an initially spherical vesicle as a function of translocation fraction ρ for a vesicle-pore system simulated using triangulated membranes with $R_\mathrm{p}/R_\mathrm{v} = 0.5$, $\kappa_\mathrm{v}/\kappa_\mathrm{h} = 1$ and $\gamma = 0$. The vertical dashed lines indicate the range of translocation fractions where the vesicle touches the rim of the pore.

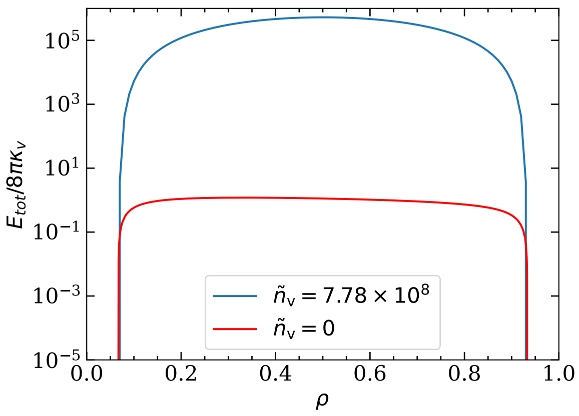

FIG. S10: Energy barriers created during translocation of an initially spherical vesicle of volume 21 μm^3 as a function of the translocation fraction ρ for a vesicle-pore system simulated using our spherical-cap model with $R_\mathrm{p}/R_\mathrm{v} = 0.5$, $\kappa_\mathrm{v}/\kappa_\mathrm{h} = 1$ and $\gamma = 0$ for osmotic concentrations $c_0 = 308 mOsm/\ell$ and 0.

states exist for $v = 0.8$ prolate vesicles. I.e., stable shallow-translocated states are missing for all bending-rigidity ratios, see Fig. S8(b), as well as for vanishing host-membrane tension, see Fig. S8(c). Furthermore, the parameter regime for deep translocated states is larger for $v = 0.6$ compared to $v = 0.8$, which reflects the higher energy barrier to the increased membrane curvature at the vesicle tips.

S9. VOLUMES OF INITIALLY SPHERICAL VESICLES DURING TRANSLOCATION

Figure S9 shows the volume of an initially spherical vesicle with fixed membrane area for various translocation fractions. Before the vesicle touches the rim of the pore, the volume decreases only slightly. After touching, the pore directly constricts the vesicle shape, and the volume decreases strongly with a minimum of around half translocation. Finally, after detaching from the rim of the pore, the vesicle volume increases until it recovers its initial volume at complete translocation.

S10. ENERGY LANDSCAPES AT FINITE OSMOTIC CONCENTRATIONS

Vesicle translocation driven by adhesion to a host membrane and by an osmotic-pressure difference across a pore are qualitatively different. In the first case, a contact energy between the vesicle and the host membrane drives the translocation, and–in addition–the deformation energy of the host membrane needs to be considered. The adhesion-energy gain is proportional to the translocated vesicle area. In the latter case, the pressure difference across the pore drives vesicle translocation, and the energy gain is proportional to the translocated vesicle volume. However, further aspects must be considered at finite osmotic concentration, particularly for the translocation of initially spherical vesicles.

Figure S10 shows energy barriers for pore-translocation of an initially spherical vesicle with volume $21\,\mu m^3$, corresponding to the volume of a Toxoplasma gondii tachyzoite [2], at physiological osmotic concentration $308\,mOsm/\ell$ through a pore of radius $R_\mathrm{p} = 0.86\,\mu m$ ($R_\mathrm{p}/R_\mathrm{v} = 0.5$). This corresponds to 7.78×10^8 solute particles inside the vesicle, and the osmotic-pressure energy contribution [3]

$$E_\mathrm{p} = \tilde{n}_\mathrm{v}(v - \ln v - 1)k_\mathrm{B}T \tag{S1}$$

to the vesicle deformation energy, where $\tilde{n}_\mathrm{v}$ refers to the number of solute entities enclosed in the vesicle and v its reduced volume, which changes during translocation, compare Fig. S9. We assume that the osmotic-pressure difference between the vesicle's interior and exterior vanishes for the undeformed, spherical vesicle shape. The osmotic-pressure contribution to the deformation energy of an initially spherical vesicle at physiological osmotic concentration results in a translocation-energy barrier five orders of magnitude higher than a typical barrier caused by membrane deformation energies. This may suggest why parasites have reduced volumes $v < 1$, requiring no volume changes during invasion.

Figure S11 shows energy landscapes for both translocation mechanisms at the pore-passage transition for $R_\mathrm{p}/R_\mathrm{v} = 0.5$, $\kappa_\mathrm{v}/\kappa_\mathrm{h} = 1$, and $\gamma = 0$, i.e., assuming that the barrier is caused by membrane bending only. The barrier heights are similar, slightly higher for the osmotically driven system. For the osmotically-driven system, the shallow-translocated state is not stable. Although the discussion for an initially spherical vesicle without considering the osmotic-pressure energy barrier serves the comparison of the driving mechanisms and is

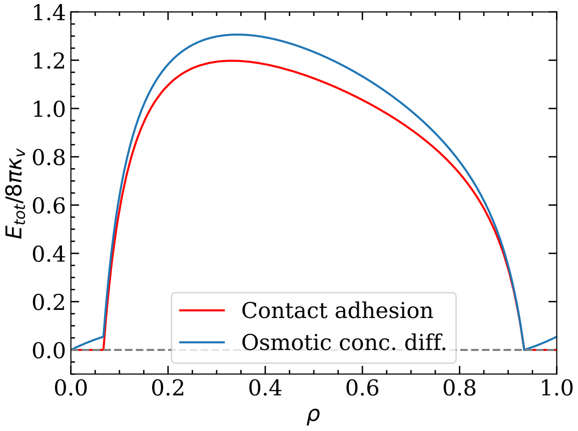

FIG. S11: Energy barriers created during translocation of an initially spherical vesicle for a vesicle-pore system simulated using our spherical-cap model with $R_\mathrm{p}/R_\mathrm{v} = 0.5$, $\kappa_\mathrm{v}/\kappa_\mathrm{h} = 1$ and $\gamma = 0$ for driving forces created by a contact adhesion between the vesicle and the pore-spaning membrane at $\tilde{w} = 1$ and an osmotic concentration difference, $\Delta\tilde{n}_v = 4,742$ across the pore.

not directly comparable to experimental systems, it provides an estimate for the required pressure difference to drive translocation. The osmotic concentration difference required for the free state I to have the same energy as the deep-translocated state III is $\Delta\tilde{n}_v = 4,742$. Assuming an initial vesicle of volume $21\,\mu m^3$ (similar to free Toxoplasma gondii), this corresponds to an osmotic-concentration difference 3.75×10^{-4} $mOsm/\ell$. Note that this value is much smaller than the physiological osmotic concentration and also the osmotic concentration differences $\Delta c = 415$ $mOsm/\ell$ in human skin sections [4]. However, our estimate is similar to the concentration differences reported for *in vitro* experiments with vesicles [5], where $\Delta c = 10^{-4}$ $mOsm/\ell$.

S11. PRESSURE AND TENSION OF PROLATE VESICLES DURING TRANSLOCATION

For the initially prolate vesicles, the total surface area and the reduced volume of the vesicles are kept constant at their target values throughout the pore translocation. This requires finite values of the Lagrange multipliers that change during translocation. Figure S12(a) shows the pressure difference p_v between the prolate vesicles and their environment for various translocation fractions, which is the multiplier for the V_v in Eq. (1). For $v = 0.6$, the initially negative pressure decreases further with increasing vesicle translocation, as the deformation-energy costs for the adhered host membrane aim to shrink partial-translocated vesicles. For $v = 0.8$, the initially negative pressure becomes positive and shows a peak near half-translocation at $\rho = 0.5$; this coincides with the translocation fraction where the vesicle is constricted most and–without a finite the Lagrange multiplier–would

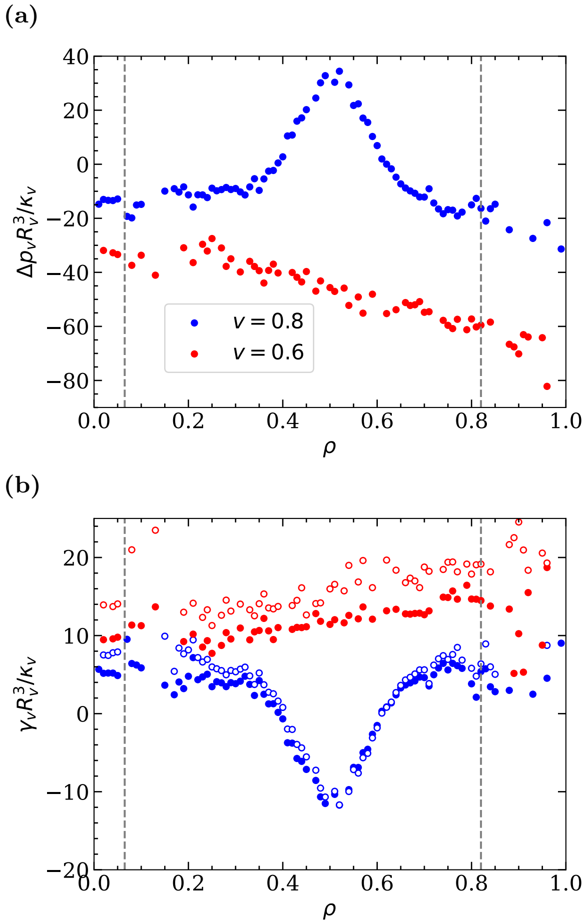

FIG. S12: Lagrange multipliers for prolate vesicles with $v = 0.8$ and $v = 0.6$, and $R_\mathrm{p}/R_\mathrm{v} = 0.5$, $\kappa_\mathrm{v}/\kappa_\mathrm{h} = 1$ and $\gamma = 0$. (a) Pressure differences between the interior of the vesicle and the environment for various translocation fractions. (b) Membrane tensions for various translocation fractions. The filled and open symbols represent the tensions in the free and adhered vesicle membranes, respectively. The vertical dashed lines, as in Fig. 2, indicate the range of translocation fractions where the vesicle touches the rim of the pore.

assume its minimal volume, compare Fig. S9.

Figure S12(b) shows the membrane tension γ_v for various translocation fractions, which is the Lagrange multiplier for the membrane area S_v in Eq. (1). Here, the tensions for the free and adhered vesicle areas are reported separately because of the target area translocation fraction for that both areas are separately kept fixed for each calculation. The tension is positive for free vesicles and shows negative values and a peak for half-translocated vesicles with $v = 0.8$. The $v = 0.6$ vesicle is thinner and does not deform as it translocates; therefore, the tension monotonically increases near half translocation.

[1] U. Seifert, Adv. Phys. **46**, 13 (1997).
[2] E. R. Firdaus, J. Park, S. Lee, Y. Park, G. Cha, and E. Han, J. Biophotonics **13**, e202000055 (2020).
[3] Q. Yu, S. Dasgupta, T. Auth, and G. Gompper, Nano Lett. **20**, 1662 (2020).
[4] G. Cevc and G. Blume, Biochim. Biophys. Acta, Biomembr. **1104**, 226 (1992).
[5] G. T. Linke, R. Lipowsky, and T. Gruhn, Europhys. Lett. **74**, 916 (2006).
